# Review of Collars, Harnesses, and Head Collars for Walking Dogs

**DOI:** 10.3390/ani15152162

**Published:** 2025-07-22

**Authors:** Camila Cavalli, Alexandra Protopopova

**Affiliations:** Animal Welfare Program, Faculty of Land and Food Systems, The University of British Columbia, Vancouver, BC V6T 1Z4, Canada; a.protopopova@ubc.ca

**Keywords:** dog walking, leash, collar, head collar, head halter, harness, martingale collar, restraint devices

## Abstract

As dogs are often required to be leashed in public, guardians need to choose between various restraint devices. While using collars typically considered aversive (such as choke, prong, or electric collars) is generally discouraged due to welfare concerns, guidance is less clear when it comes to selecting among other devices such as collars, harnesses, and head collars. This review examined 21 full-text articles and two abstracts on the effects of commonly used restraint devices, aiming to offer practical guidance for guardians and identifying areas for future research. The impact of these devices was examined in terms of walking kinematics, pressure distribution on the body, and behavioural signs of stress. The findings suggest there is no one-size-fits-all device, and selection should consider the individual needs of guardian and dog. For dogs that pull, non-tightening front-clip harnesses appear to offer the best balance between discomfort and reduction in pulling. Tightening harnesses, martingale collars, and head collars can pose greater discomfort and should be used with caution. For brachycephalic breeds or when pulling is not a concern, back-clip harnesses are suitable, especially chest-strap or Y-shaped ones. Flat collars are also appropriate for dogs that do not pull as they produce the least body restriction.

## Simple Summary

Dogs are often required to be on leash, but choosing between different types of restraint devices, like collars, harnesses, and head collars, is not always straightforward. Previous research has advised against the use of aversive collars (such as choke, prong, or electric collars); however, guidance is less clear on how to choose between other types of restraint devices. This review looked at existing research to better understand how these other devices can affect dogs’ movement, how pressure is distributed on their bodies, and whether dogs show signs of stress while wearing them. The results showed that no single device works best for every dog, and the right choice depends on the dog and the guardian’s needs. For dogs that pull, non-tightening front-clip harnesses seem to offer a good balance between discomfort and prevention of pulling. Tightening harnesses, martingale collars, and head collars may be less comfortable and should be used with caution. For brachycephalic dogs, or in cases where pulling is not a concern, back-clip harnesses are suitable, especially those with a chest-strap or a Y-shape. Flat collars are appropriate for dogs that consistently walk without pulling, as they produce the least body restriction.

## 1. Introduction

Dogs are one of the most common companion animals worldwide, and their popularity has been rising steadily over the past decades [1,2]. While globally, a substantial proportion of the dog population is not under human control [3], in many Western countries, dogs are not allowed to roam in public spaces [4]. This is often due to concerns about reliable recall and risks of injury, for example, due to proximity to traffic, protection of wildlife and farm animals, prevention of biting incidents, dog waste and damage, and the enforcement of leash laws in many public areas [5,6,7]. Therefore, many guardians choose to walk their dogs while the dog is restrained by a leash. Dog walking is thought to have originated in the 19th century, allowing dogs to access public spaces in a controlled manner [8] and is often regarded as a fundamental component of responsible dog ownership, providing them with exercise, mental enrichment, and socialization opportunities [9,10,11]. Moreover, increased physical exercise due to dog walking has also been reported as beneficial for their guardian, and guardians who frequently walk their dogs have higher levels of attachment to them [11].

To restrain dogs on walks, leashes are typically attached to a collar around the dog’s neck, a harness around their body, or a head collar over the dog’s head. Additionally, some working dogs, such as assistance dogs, are required to wear specialized equipment designed to facilitate their role in guiding human handlers [12]. We found no research specifically outlining how guardians select dog walking equipment. One consideration may be selecting equipment that can reduce the dog’s pulling on the leash. This is a frequent behavioural concern, with guardian-reported survey data suggesting prevalence of leash-pulling as high as 69% in the UK [13] and even rising to 82% in a more recent survey conducted in the UK and Ireland [10]. Guardians reported that the perception of being unable to control the dog made the walk less pleasurable [11]; and, in fact, poor control of the dog may even lead to physical injuries to guardians due to falls and strain of the arm and shoulder [14,15,16]. As a consequence, pulling can lead to decreased quality, frequency, and duration of walks. And in turn, reduced exercise could lead to further problems, such as weight gain and decreased mental stimulation for the dog, which may contribute to the development of other undesirable behaviours [6].

Dog equipment can be loosely divided into devices that are typically considered ‘aversive’ and those that are not typically considered aversive. While any device could be potentially aversive if it is perceived by the dog as uncomfortable, items that are generally regarded as aversive are intentionally designed to inflict pain or discomfort to punish certain behaviour [17,18]. For collars that are typically considered aversive (such as electronic, prong, and choke collars), previous research has repeatedly demonstrated associations with negative effects on animal welfare and decreased training success [19,20] (for reviews, see [17,18,21]). Because of this, their use has been discouraged by many animal welfare, training, and veterinary organizations (e.g., American Veterinary Society of Animal Behavior (AVSAB) [22]; Australian Veterinary Association (AVA) [23]; Canadian Veterinary Medical Association (CVMA) [24]; Certification Council for Professional Dog Trainers (CCPDT) [25]; Pet Professional Guild (PPG) [26]).

Less is known about any effects of restraints that are typically not considered aversive such as collars, harnesses, and head collars, and guidance for guardians is limited. Guidelines mostly compile descriptive information, and when recommendations are provided, they seem to reflect observations mostly grounded in common sense, usually presented without supporting evidence. For example, both the American Kennel Club [27] and Humane World for Animals [28] describe the features of different types of collars and the importance of a proper fit, but do not provide many recommendations about choosing equipment. The limited recommendations include HWA’s statements that martingales (i.e., neck collars that have a loop which can tighten up to a point) are a humane option for dogs likely to slip out of a regular collar; and head collars may be helpful for strong, energetic dogs likely to jump [28]. Other recommendations highlight broader issues such as VCA Canada’s mention of the risk of entanglement [29]. Alternatively, some recommendations reflect the perceived potential for physical or welfare harm that the equipment may cause. For example, the AKA recommends using harnesses in the case of brachycephalic dogs, dogs with neck pain, breathing issues, or heart disease, as well as for dogs that pull on the leash [30]. In the same line, the popular pet supplies company PetSmart [31] recommends using a harness in the case of puppies, small dogs, and dogs with neck issues, but cautions that dogs may be more likely to pull when wearing one, and suggests consulting with a veterinarian for advice on choosing a device and ensuring a proper fit. Lindell et al. [29] from VCA Canada mention a series of benefits and drawbacks of different types of restraints, highlighting potential issues such as pressure on the neck or the joints. However, in these latter cases, supporting evidence about the prevalence and mechanism of harm or the specifics of equipment features to avoid is not provided.

For all these reasons, it is important to assess available research related to restraining dogs during walks, translate the findings into practical recommendations for dog guardians, and identify areas for future research. Research examining the effects of restraint devices which are typically not considered aversive will be reviewed, with a focus on outcomes related to walking kinematics, leash tension and pressure on the body, stress, behaviour, and overall welfare. Investigating the effects of various restraint methods is essential to better understand humane handling practices as well as identify potential welfare concerns, particularly for dogs that pull. Such research can inform evidence-based recommendations, contribute to policy development, and guide guardians in selecting appropriate equipment for their dogs.

## 2. Materials and Methods

### 2.1. Article Selection

Articles were selected through keyword searches conducted in Google Scholar in February and March of 2025. Search terms included: “dog harness”, “dog collar”, “dog head collar”, “dog restraint methods”, “dog walking”, “collar injury”. The number of search results was not systematically recorded; instead, studies were selected based on relevance to the potential health and welfare implications of using restraint devices. The reference lists and citing works of included articles were also examined to identify additional relevant studies. To be eligible for inclusion, studies were required to have at least an abstract available in English and feature some type of assessment of the impact of wearing restraint devices in dogs. Studies focussing exclusively on shock, prong, and choke collars were removed. A total of 21 full-text articles and 2 abstracts were included.

### 2.2. Definition of Restraint Methods

The following definitions have been created to facilitate comparisons across studies, as different terms are often used to refer to similar devices.

Note that while most of these restraints are not typically considered aversive in the same way that shock, prong, and choke collars are, some of them may be perceived as aversive by the dog as they incorporate stimuli that are physically or emotionally uncomfortable [18]. This will be further addressed in the Discussion section.

Collar: Also called “flat collar” and “neck collar”. A strip of fabric (typically leather or nylon) that fits around a dog’s neck.

Martingale collar: Also called “limited slip collar”. Commonly used with sighthounds and other dogs with narrow heads which may have an increased risk of slipping out of a regular collar. It can be made entirely of fabric or from a combination of fabric and chain. This collar tightens under pressure, but only up to a predetermined stopping point. This means that it differs from a choke collar because it is designed not to tighten indefinitely. However, it is important to note that the adjustable larger loop could be fitted in a variety of ways: if kept loose on the neck, it cannot constrict more than a flat collar would when pulled; if kept tight, it would function as a choke collar rather than a martingale.

Head collar: Also called a “head halter”. This collar has a strap around the dog’s muzzle below the eyes which connects with a strap that sits high at the neck. Depending on the model, it may have the attachment point at the dog’s chin or at the back of their head. Some models are designed to fit tightly around the muzzle, while others only tighten when pulled.

Harness: May be constructed using straps, fabric, or mesh. Fits around the body, chest, and shoulders. Note that harnesses of different shapes may have different attachment points for the leash, and some may include both front and back clips.

According to design features:

- Chest-strap harness: Also called “straight front”, “Norwegian harness”, and “restrictive harness”. Has a strap that runs horizontally across the chest.
- Chest-plate harness: Has a panel across the dog’s chest, connected by adjustable straps around the neck and torso. When worn, it looks similar to a Y-shape harness, so they are sometimes grouped together.
- H-shape harness: Has one strap around the neck and one around the chest, connected by a vertical strap along the back.
- Step-in harness: It is slid up the legs and then fastened at the back. Does not require putting it over the dog’s head.
- Tightening harness: Designed to tighten when the dog pulls. These are usually marketed as a “training aid”, and sometimes their websites promote their suitability for breeds which cannot wear headcollars, such as brachycephalic dogs. These include harnesses with a figure eight shape, harnesses that tighten either at the front or the back under pressure, as well as the use of special leashes that loop around the torso.
- Y-shape harness: Also called “non-restrictive harness”. Has a strap that runs down the chest and between the front legs.

According to the clip location:

- Back-clip harness: Has the attachment point at the back, close to the shoulders.
- Front-clip harness: Has the attachment point at the front, close to the sternum. Some of these harnesses are also called “no-pull harnesses”.

### 2.3. Methods of Measurement

The impacts of restraint devices have been explored through various approaches, including studies focusing on walking kinematics, the effects of increased pressure on the body, and behavioural indicators of stress and discomfort.

Kinematic evaluation assesses gait by objectively describing joint angles, which can be measured using two- or three-dimensional models [32]. These studies aim to determine whether restraint methods compromise normal movement patterns, as restricted or unnatural positioning could lead to repeated strain and increase the risk of musculoskeletal injuries over time. This area of research is also informed by studies examining gait symmetry in both healthy dogs and those exhibiting lameness, assessing compensatory mechanisms such as increased use of contralateral limbs to offset impaired function [33,34]. In line with this, some studies found that the side on which the handler walks accounts for variations in limb symmetry while walking [33,35], so the handler side has been reported in Supplementary Tables S1–S6 whenever available.

To assess gait patterns, as well as the movement of the limbs, these studies typically use tape markers that are located on key anatomical areas. For the thoracic limbs, some of these reference points are the metacarpal bone, the ulnar styloid process, the lateral epicondyle of the humerus, the greater tubercle of the humerus, and the dorsal aspect of the scapular spine. For the pelvic limbs, these landmarks include the distal lateral aspect of the fifth metatarsal bone, the lateral malleolus of the fibula, the lateral femoral condyle, the greater trochanter of the femur, and the iliac crest. In the case of the spine, relevant areas include the sacral apex, the dorsal spinous process of vertebra L7, the dorsal spinous process of vertebra T13, the dorsal spinous process of T1, and the occipital protuberance in the case of the spine [36,37,38]. Common gait measures include step length, stride length, step width, body weight distribution across the paws, and the room of movement of the limbs [37,39]. These measures can be evaluated across the sagittal plane (flexion-extension), the transverse plane (internal-external rotation), and the frontal plane (abduction-adduction) [40].

Some studies assessed the walking kinematics of dogs walking on the ground, while others measured walking using a treadmill. Torres et al. [41] observed that data collected from ground and treadmill-based ambulation were comparable for dogs but also noted some differences in gait according to each mode. Moreover, studies using a treadmill often incorporate pre-training sessions to get the dogs used to the apparatus, which is time-intensive and may lead to the exclusion of potential subjects. While Torres et al. [41] highlighted that habituation to the treadmill occurred rapidly in their case, Lafuente et al. [32] indicated that almost 30 dogs started in their study, but only 9 ended up completing all testing procedures. Despite these potential limitations, reliability and reproducibility have been cited as reasons to justify the use of a treadmill in studies of gait analysis [42].

Other studies have assessed detrimental effects by measuring restraint pressure. For instance, pressure on the neck may damage tissue, restrict blood circulation to the brain, and compromise the airway by obstructing airflow [43]. It has been noted that even small amounts of pressure can cause injuries, especially those applied over a sufficiently long period of time in areas poorly protected by soft tissue or fur [44,45]. Although capillary pressure can vary considerably between species and among individuals within a population, reduced blood flow is generally considered harmful. This is because it limits the delivery of oxygen and nutrients to tissues while allowing waste products to accumulate, potentially leading to tissue damage and impaired function [44]. Pressure values will be reported in kilopascals (kPa), a standard unit commonly used in biomechanics. Conversions from Newtons per square centimetre (N/cm^2^) or millimetres of mercury (mmHg) will be provided when original sources report data in those units, with the original values stated in parentheses. While there is no research on the influence of pressure on the skin of dogs, in humans, deterioration of tissue can start occurring when pressure exceeds capillary pressure, which is estimated at 4.3 kPa (0.43 N/cm^2^) (Landis, 1930, as cited in [45]). Moreover, some authors measured intraocular pressure, as increased pressure on the neck can also compress the jugular veins and potentially cause elevation of this parameter [46,47]. A case study reported a dog with symptoms of hypoxia following strangulation caused by being suspended off the ground with a choke collar as a form of discipline [48]. Although this is an extreme case and involves the use of an aversive-based collar, it highlights the potential risk of injury when sudden or prolonged forces are applied to the neck. Detrimental effects of increased pressure on sensitive areas have also been observed in humans, such as elevated intraocular pressure when wearing a tight necktie [49]. Additionally, tourniquets used to stop bleeding can exert pressures of up to 33.3 kPa (250 mmHg) on the arm and 39.99 kPa (300 mmHg) on the thigh, and higher pressures have been associated with an increased risk of associated injuries [50,51]. In the case of horses, Casey et al. [52] recorded noseband pressure ranging from 26.66 to 53.32 kPa (200 to 400 mmHg), which they noted could be enough to cause nerve damage in humans.

Rein tension metres have been developed in equitation science [53], and some authors have adapted this technology to measure the force exerted through pulling the leash in the case of dogs. This is typically conducted by measuring leash tension (expressed in newtons) using tension metres or load cells attached to the restraint device or the leash. Other related measures include contact pressure, mean force, and peak force [43,45]. In some cases, researchers have also included differential measures to distinguish whether the pulling is initiated by the dog or the handler [54].

In other cases, research has turned to observing the behaviour and physiological response of dogs while wearing different restraint devices. The rationale for studying these signals is to assess whether dogs experience stress in these situations, if they seek guidance from humans, and whether they refuse to walk. In terms of stress signals, this category usually includes self-directed behaviours such as scratching, lip licking, yawning, and trembling, which are generally understood as indicators of motivational conflict and a possible by-product of a physiological stress response [55,56]. Other related indicators include the position of the ears, the tail, and the general posture of the dog, including lifting a paw, cowering or crouching, as well as the presence of vocalizations such as barking or whining [57,58,59,60]. In addition to assessing behavioural indicators of stress, one study also measured related physiological parameters including cortisol levels, blood pressure, and heart rate [60]. Other commonly analyzed behaviours include human-directed behaviours, such as gazing at the person holding the leash [57,59,60]. Finally, some studies incorporated measures related to refusal to walk (i.e., baulking), as well as attempts to remove the restraint method in the case of head collars [60,61].

## 3. Results

Comparisons of different types of restraint devices will be presented in this section, providing a detailed analysis of each category. See Supplementary Tables S1–S6 for an overview of methodological aspects and key findings of each study, including sample details, devices tested, and general procedures; and Supplementary Table S7 for a summary of findings.

### 3.1. Comparison of Collars

The following studies focused on comparing the effects of different types of collars (see Supplementary Table S1). Hunter et al. [45] examined the pressure and force applied by three types of collars on eight dogs of various breeds. The examined collars included a “double layer polyester and nylon weave” (i.e., a padded collar), a “single layer nylon weave”, and a “single layer canvas strip”. Pressure was measured using a device placed ventrally between the collar and the neck of the dog. All dogs were led by the same person while walking in straight, clockwise, and counterclockwise directions. The direction of the exercise affected the amount of pressure and force applied to the neck, as the mean force was higher when walking in circles compared to walking in a straight line. The authors highlighted that contact pressure reached 45.8 kPa (4.58 N/cm^2^), which was greater than what was observed in horses using ill-fitting saddles at 38.9 kPa (3.89 N/cm^2^) [62]. In terms of peak force, the average was 302 kPa (30.2 N/cm^2^), which was comparable to the maximal force of 303 kPa (30.3 N/cm^2^) observed by Peham et al. [12] in the case of guide dogs wearing a harness (see details below), dogs pulling a wheelchair (293 kPa [29.3 N/cm^2^]) or a sled (267 kPa [26.7 N/cm^2^]) [63]; as well as to what has been observed in horses ridden with a bit [42]. Nevertheless, it should be noted that the absolute maximal force observed was significantly higher at 730 kPa (73 N/cm^2^). Considering the dog’s skin is thinner ventrally where collar force is applied, the authors highlighted the potential risks of such elevated force on this sensitive area.

Comparing the types of collars, results showed that the double weave (i.e., padded) collar exerted the highest contact pressure as well as the highest peak force, evidencing a poor distribution of force across the neck. This suggests that the cushioning in the double weave collar was not only ineffective in reducing force, but it instead concentrated pressure into a smaller area, potentially increasing the risk of discomfort or injury. This unexpected outcome seems to be contrary to the guardian’s likely expectations when selecting a padded collar and could potentially be due to the cushioning not being present around the whole circumference of the collar, creating a reduced contact area.

Carter et al. [64] explored the pressure exerted by a light pull, a strong pull, and a jerk using different types of collars on a simulated neck model fitted with a pressure sensor. They selected seven collars and a slip lead that differed in material (nylon, leather, or metal), thickness, and padding. They observed a great variability in the pressure exerted by the collars, which ranged from 83 kPa to 832 kPa. Nevertheless, they noted that even the lowest value was much higher than values that can cause increased intraocular pressure, tissue damage, and necrosis in humans, where values as low as 4.3 kPa have been flagged for concern [51,52]. Moreover, it has been suggested that saddle pressure greater than 4.67 kPa could cause damage due to a reduction in skin perfusion in horses, while other authors have indicated that values above 11 kPa could cause back pain, besides the risk of ischemic injuries [62]. Based on these data, the authors concluded that all of the tested collars could potentially generate enough pressure to cause injury in dogs, regardless of the type of collar.

Nevertheless, there were some relevant differences between the collars. As the force increased (i.e., a strong pull), the padded webbing collar, rope slip lead, and lurcher collars were the ones that exerted the lowest pressures. Furthermore, there were differences in the areas of contact and the distribution of pressure among the collars. The slip lead and chain collars concentrated the force on a smaller area compared to a wide leather collar. In relation to this, the authors noted that a smaller contact area reduces the distribution of pressure and focuses the force on a limited area, increasing the likelihood of injury. Additionally, the chain collar and the leather and thread collar exerted more pressure on the middle of the neck rather than the sides. The padded webbing collar went from causing a relatively even pressure with the light pull to a higher pressure in the middle of the neck with the stronger pulling scenarios, which the authors attributed to the padding not covering the entire collar in line with Hunter et al. [45] observations. While a smaller surface area and pressure in the middle of the neck could pose a greater risk, the authors emphasized that no collar could completely prevent the risk of injury if the dog pulled, questioning the suitability of using any collars for these dogs.

### 3.2. Comparison of Harnesses

Supplementary Table S2 lists the studies that primarily compared different types of harnesses (note that some included collars as a baseline measure).

Kiss et al. [37]’s research report analyzed how different types of harnesses affected walking kinematics. The three harnesses used were all manufactured by Julius-K9^®^, custom-made not to include any light reflective materials that could affect the motion capture measurement. Dogs were encouraged with treats to maintain a continuous walk on a treadmill or to walk across the room towards their guardian calling them. Gait kinematics were assessed using a 3D motion capture system which detected reflective markers placed on key anatomical points. Measurements included step length, stride length, step width, and height of the thoracic and pelvic limbs. Leash force was measured using a load cell at the end of a retractable leash. Walking kinematics differed between the treadmill and the ground, probably due to the forced speed on the treadmill. However, there were no significant differences across harnesses nor when comparing them to walking without a harness. When force was applied with the leash, dogs switched to a slower gait pattern when walking on the ground, but this effect was similar for each harness.

Pálya et al. [65] appeared to include part of the data reported in Kiss et al. [37]. They developed a detailed gait analysis method to examine how different harnesses affected walking kinematics compared to free movement without restraint. This pilot study included four dogs of various sizes which were trained to walk on a treadmill. Dogs were observed walking without a harness, then walking with different harnesses without a leash, and then with different harnesses attached to a leash. Two of the harnesses tested were regarded as “restrictive” (chest-strap harness), while the other was considered “non-restrictive” (Y-shaped harness). Reflective markers were placed on anatomical landmarks across the body, and measurement was carried out with a 3D optical motion capture system. The authors concluded that this type of gait analysis was successful in exploring an extensive array of parameters, and this methodology could be applicable to various dog sizes and harnesses. In terms of gait patterns, they indicated that leashed walks were the most different from the baseline free movement observation. However, as all harnesses altered the gait patterns of the dogs, the authors highlighted that there does not seem to be a single option that is universally superior. They concluded that more research is needed to determine which harnesses are better suited on a case-by-case basis, considering factors like the size of the dogs and the context of use, such as walking in the city compared to hiking.

Lafuente et al. [32] compared the effects of two types of back-clip harnesses on shoulder movement in nine dogs while walking and trotting. They tested what they named a “non-restrictive” (Y-shaped) harness as well as a “restrictive” (chest-strap) harness. They based their categories on the presumed limiting effect of the harnesses on the range of motion of the shoulder due to the strap running across the chest. Additionally, they also included a condition with 2.5 kg weights added to simulate pulling from the leash. Dogs were first habituated to walking on a treadmill to ensure constant walking and trotting speed, then tested in the following fixed order: no harness, Y-shaped harness, Y-shaped harness plus weights, chest-strap harness, and chest-strap harness plus weights. The results indicate that every harness condition restricted shoulder extension significantly more than not wearing a harness. Surprisingly, the Y-shaped harness (which was considered non-restrictive) limited shoulder extension more than the chest-strap (“restrictive”) harness, with and without weights. The addition of weights significantly affected shoulder extension during walk but not trot in the Y-shaped harness, while it had no significant effects in the case of the chest-strap one. The authors concluded that the shape of the harnesses had a bigger effect on shoulder extension restriction than the application of resistance with the weights.

Wiliams et al. [38] studied the effects of three types of harnesses on walking in 66 dogs of five popular breeds (Cocker Spaniels, Springer Spaniels, Labrador Retrievers, Staffordshire Bull Terriers, and French Bulldogs), as well as mixed-breed dogs. The assessment included two Y-shaped harnesses and a chest-strap harness. Dogs were also assessed wearing a collar, and their usual harness if they wore a different model. A pressure-sensing mat was used to examine the gait of the dogs while they walked on a loose leash clipped at the back and held by their guardians. Measures included stride length, percentage of weight distributed across the front and the back paws, and angulation of the humerus during the motion cycle. Tests were videotaped, and the recordings were used to calculate the elbow-to-floor distance for each dog. Stride length was calculated as a proportion of the elbow-to-floor distance to allow comparisons between dogs.

There were differences in stride length, body weight distribution, and estimated angulation, but variations were not consistent for each harness and highly differed across breeds. The authors concluded that all harnesses had the potential to alter canine gait. In line with previous research, they indicated that no harness appears to be superior for all dogs, and fitting a harness should be performed on an individual basis with special consideration to the dog’s anatomy. For instance, they noted that the distance from the floor to the elbow appeared to be a key factor in determining whether a harness restricted stride length. They observed that in breeds with relatively longer limbs compared to their overall height (such as Retrievers or Spaniels), the chest strap may end up sitting at a position that is not as restrictive as it is generally thought. However, they also indicated that this may reduce the efficacy of the harness in decreasing pulling and highlighted that further research should include conditions in which dogs pull. In a similar way, the fit of the harness in relation to the shoulder blade appeared to impact body weight distribution, but only in some of the tested breeds. Future studies should also consider specific factors within each harness such as the width of the straps and the presence of padding.

Dowdeswell and Churchill [66] tested 30 dogs of various sizes while walking on leash wearing six types of harnesses compared to a collar. Measures included shoulder and elbow extension and flexion. The results indicate that using a Y-shaped harness clipped at the front and no-pull harnesses had the most impact on elbow extension, followed by the chest plate and step-in ones. Conversely, there were no differences in the chest-strap or the H-shaped harnesses compared to the collar. In terms of elbow flexion, the most impact was observed when using the front clip on the Y-shape harness and step-in harnesses, while the chest-strap, H-shaped, and chest plate harnesses did not impact this measure. Regarding shoulder movement, all harnesses significantly impacted shoulder extension compared to the collar. Additionally, the chest plate, front clip Y-shaped, step-in. and H-shaped harnesses had the greatest impact on shoulder flexion, while the chest-strap one had no significant impact. Although more research is needed, and poor fit is an ongoing concern that can increase the negative impact of any restraint method, the authors made some recommendations based on their results. Specifically, they highlighted that using the front-clip on a Y-shaped harness significantly reduced all four measures, which raised concerns about its suitability. Conversely, they recommended the chest-strap harness, which did not affect three out of the four measures despite its reputation as being restrictive, as well as the H-shaped one.

### 3.3. Comparison of Collars and Harnesses

Some studies focused on comparing collars and harnesses; see Supplementary Table S3 for details.

Pauli et al. [47] examined the effects of wearing a harness or a nylon collar on intraocular pressure in 26 sled dogs. All dogs had been trained to pull on a tether on command in preparation for sled pulling. A force gauge measured the tension in kilograms each dog generated against the leash when wearing a collar or a harness. Then, this tension was replicated by pulling on a leash when the dog was restrained while wearing either restraint method. Intraocular pressure was measured as a baseline before pulling, 10 s after pulling, and 1 min after pulling. The results exhibit a significant increase in intraocular pressure from the baseline when wearing a collar (51.6% increase), but not when wearing a harness (15.8% increase). Moreover, intraocular pressure increased by a mean of 0.98 kPa (7.4 mmHg) with a collar and 0.30 kPa (2.3 mmHg) with a harness. After 1 min, intraocular pressure returned to baseline in both conditions. The authors noted a trend towards greater intraocular pressure as the age of the dog increased, but it did not achieve statistical significance. In terms of breed, Alaskan malamutes and Siberian Huskies experienced smaller changes in intraocular pressure than the other dogs. The authors recommended that dogs with conditions that may be aggravated by increased intraocular pressure (e.g., thin corneas, glaucoma) wear a harness, especially during exercise.

Grainger et al. [57] focused on behavioural indicators of stress when dogs were walked on a collar or a harness. They tested a total of 30 dogs, half of which were regularly walked using a collar, and half using a harness. On the first visit, dogs were walked by their guardians for 20 min on a 1 m leash using their usual restraint method. Afterwards, they were given a fleece-lined collar or a harness and invited to return for another walk after one week of habituation to the new device. Video recordings of the walks were used to examine potential stress indicators such as licking lips, yawning, low body position, low tail position, ears held low or pulled back, vocalizations, paw lifting, panting, and trembling/body shaking, as well as looking at the guardian and other indicators of restraint (sniffing the ground, tracking, stopping). Most stress indicators had a low frequency in both conditions. There were moderate frequencies of panting and looking at the guardian, as well as high frequencies of lip licking, but these did not differ across conditions. No significant differences were found between the restraint devices or in terms of previous history using either device, except for the case of ear position. Specifically, dogs with a history of wearing a collar had a higher frequency of having their ears back, but there were no significant effects of the type of restraint used in the test. While this could indicate increased stress in dogs regularly walked on a collar, this finding should be considered with caution as it is not supported by other stress measures. The authors concluded that dog welfare was not compromised by either restraint type, at least for the specific harness and collar trialled in this study.

Zilocchi and Parisi [67] set out to investigate the effects of wearing a collar or a harness while meeting an unfamiliar dog. The study included 18 dogs who were walked on a 1.5 m leash fixed to a nylon collar or a harness (model not specified). Dogs were used to wearing both types of restraints. Encounters with an unfamiliar dog were carried out on leash in a fenced area and lasted 1 min. Several potential stress indicators were examined, but differences between restraint methods were minimal. Specifically, dogs exhibited more nose licking when wearing the harness, and more paw lifting when using the collar. Moreover, dogs exhibited more attention-seeking behaviours towards the guardian when wearing the collar (note that while the article mentioned all the behaviours that were measured, it did not specify which ones were considered “attention-seeking” signals). Overall, the authors concluded that neither device appears to significantly influence communication between dogs.

Shih et al. [59] compared the effects of wearing a collar or a chest-strap harness clipped at the back in 52 shelter dogs. A tension metre was secured to a wall and attached to a leash held by an experimenter, which was connected to the collar or harness while the dog was attracted with treats or toys. Measures included maximal and mean leash tension as well as pulling time initiated by the dog, which was defined as the time dogs pulled on the leash with force over 1% of their bodyweight. The medial maximal and mean leash tension, as well as the proportion of pulling time, were significantly higher when dogs were attracted with treats while wearing the harness. Dog behaviour was also coded for looking at the experimenter as well as potential stress-related behaviours including ear and tail position, panting, lip licking, sniffing, shaking, paw lifting, and vocalizations. There were no significant differences in terms of stress-related behaviours between the harness and the collar, but dogs looked more frequently at the experimenter in the food condition when wearing the harness. This may be due to dogs looking referentially at the human manipulating the food, as well as the harnesses being less restrictive of their head movement. The authors concluded that these results support the hypothesis that back-clip harnesses are associated with increased pulling on the leash.

Bailey et al. [43] compared the pulling force generated by 28 dogs of different sizes when wearing either a flat nylon collar or a padded Y-shaped harness. Dogs were presented with stimuli that could be encountered during a walk (an unfamiliar dog, food, and a thrown toy) while walking on a circuit, once wearing the collar and once wearing the harness. Measures included mean pulling force, peak force, and leash tension. The results indicate a greater mean pulling force when dogs were wearing a harness compared to a collar. In terms of the mean peak pulling force, this was also greater in the harness compared to the collar, although the highest individual peak force was registered in a dog wearing a collar. There were also some effects related to the size of the dogs. As expected, peak force increased as the size of the dogs increased. However, when force was analyzed as a percentage of the body weight of the dog, the authors observed that small dogs exhibited higher mean pulling forces than medium or large dogs. Surprisingly, peak force observed in small dogs reached 122.2% of their body weight, when only 85.8% and 82.7% were observed for medium and large dogs.

The authors concluded that using a harness resulted in an increase in pulling. They suggested that dogs may pull more on a harness because they experience less discomfort when they inflict pressure to the chest compared to the cervical area, which is likely to be more sensitive. They noted that the force distributed with a padded harness such as the one used in the study is dissipated over the sternum, resulting in a large affected area. However, they also raised concerns over the use of collars, as some dogs pulled with a force that exceeded 80% of their body weight.

Johnson and Wynne [58] compared different restraint methods in terms of their efficacy in reducing pulling and welfare impact in 23 shelter dogs. They used a martingale collar as the baseline and compared it to a front-clip harness as well as two types of prong collars (polymer and metal), which were tested in that fixed order to account for potentially increasing levels of aversiveness. Dogs were walked for 5 min using each type of equipment while a strain gauge measured their pulling force. The results showed that dogs pulled significantly more on the martingale than with all other types of equipment. The authors suggested that this may have been because the martingale was less restrictive across the shoulders and chest (compared to the harness) and inflicted less aversive pressure on the throat (compared to the prong collars). While the martingale collar was considered the baseline by the authors, an important factor to take into account when interpreting these results is that this type of collar is designed to tighten as the dog pulls, which may be perceived as aversive by some dogs. Additionally, the fixed order could be a confounding factor when analyzing this result.

Behavioural coding included several potential stress behaviours, but only lip licking, sniffing, and looking at the handler were frequent enough for statistical comparison. While there were no significant differences in frequencies of looking at the handler and sniffing across the four types of equipment, dogs lip licked more when wearing the harness compared to the martingale collar. The authors suggested this may indicate that dogs felt less constrained by the martingale than the harness, but emphasized the difficulty of making broad interpretations based on a single behavioural indicator.

Bailey et al. [46] evaluated the effects of wearing a collar or a harness on intraocular pressure and respiratory rate in 20 dogs. Half of the dogs were brachycephalic (dogs with broad heads, flat faces, and short noses and a cephalic index > 60 cm), and half were dolichocephalic (dogs with narrow heads and elongated noses and a cephalic index < 50 cm). Intraocular pressure and respiratory rate were measured immediately after five conditions: a baseline with no restraints, being stationary wearing a collar or a harness with a tense leash for 10 s, and walking on a loose leash while wearing a collar or a harness. The results indicated that collars increased intraocular pressure during exercise for all dogs, and also while stationary in the case of brachycephalic ones. This effect was not observed in the case of harnesses. Respiratory rate was elevated in brachycephalic dogs while stationary with a collar and while exercising with either device, while no differences were observed in dolichocephalic dogs. These results indicate that collars can increase intraocular pressure in all dogs regardless of their head shape, but the effects are more pronounced in dogs that naturally have higher intraocular pressure and are more predisposed to ocular pathologies. Moreover, wearing a collar also increased the respiratory rate in brachycephalic dogs. The authors noted that these dogs also had an elevated respiratory rate while wearing a harness, which suggests that harnesses may also cause pressure on neck structures in these dogs, and indicated the need for further research on the effects of harnesses with different shapes.

### 3.4. Comparison of Head Collars

A couple of studies focused on the use of head collars; see Supplementary Table S4 for detailed results.

Ogburn et al. [60] compared the physiological and behavioural responses of 26 dogs wearing a traditional nylon collar compared to a head collar consisting of tight loops of nylon going around the neck and the muzzle, with the attachment point located at the chin. They measured blood pressure, heart rate, respiratory rate, pupillary dilation, as well as plasma ACTH and cortisol levels. They also examined several behavioural measures including vocalizations, gazing at the handler, refusal to walk, pawing at the nose, and biting the leash (among others), as well as the position of the head, tail, ears, and overall posture. The tests included three repetitions of walking for 10 m and then having to sit for 10 s, until getting to the designated area for physiological measurements. The results indicated that some physiological measures decreased over time, suggesting habituation to handling and testing, but with no significant differences according to the type of restraint. In terms of behaviour, dogs wearing a traditional collar required repeated repositioning during physiological measurements, which the authors considered/regarded as a sign of disobedience. On the contrary, dogs wearing the head collar behaved in what they considered a more controlled manner, which led them to suggest that this type of collar could be an effective type of restraint. However, when wearing the head collar, dogs also showed stress signs such as lowered heads and ears as well as crouching, and they also fought the leash and pawed at their noses significantly more than while wearing a neck collar.

Haug et al. [61] compared the responses of 12 dogs to four types of head collars with slightly different designs. While all encircled the neck and the muzzle, models 1 to 3 were fitted tightly to the head, and model 4 had a looser fit. Moreover, head collars 1, 2, and 4 featured the attachment point at the chin, while number 3 had it at the back of the neck. Observed behaviours were aggregated into two groups: Group 1 included pawing, pawing at the nose, biting/pawing the leash, opening mouth, rubbing face, and shaking head; and Group 2 included rearing up, baulking, rushing forward, and rolling on the ground. Each dog was observed using all head collars, but the order was balanced according to four different sequences. Each session included three phases: 5 min where the handler was stationary to assess the dog’s initial reaction to the collar, a 2 min walking session, a 3 min period where the handler was again stationary and the leash was removed from the head collar, to test if it was possible for the dog to remove the collar by themselves. There were no significant differences among the four types of head collars for either group of behaviours. The authors concluded that there was no advantage to choosing one type of head collar over the others. However, as one dog managed to remove a collar (Response^®^), which was also one of the most difficult to fit, the authors suggested there may be some benefits in choosing the other collars. Finally, they remarked that dogs adapted quickly to the head collars despite wearing them intermittently and for short periods of time, as there was a marked decrease in both groups of behaviour across sessions.

### 3.5. Studies of Working Dogs’ Equipment

Working dogs usually wear harnesses, and in some cases need to lead people who hold these harnesses using special handles (e.g., guide dogs [12]). Taking into account both the importance of protecting the wellbeing of these animals as well as the significant investment of time and resources involved in training them, some authors have explored the potential impacts of wearing these harnesses, aiming to maximize welfare and extend working longevity. See Supplementary Table S5 for detailed results.

Peham et al. [12] examined the pressure distribution in three types of harnesses used for guide dogs. The measurements were carried out using eight guide dogs who completed a course walking in a straight line, turning left, turning right, going upstairs, and going downstairs. All harnesses were made of leather; harness 1 had loops that restricted the mobility of the frame, harness 2 was padded in the area covering the spine, and harness 3 used a rigid connection with the frame. Pressure was determined using sensor strips positioned symmetrically under the harness at key areas on both the left and right sides of the dog, including the front, sternum, chest, shoulders, and back. There were no significant differences when walking in a straight line compared to walking in a curve or using stairs. For all harnesses, the highest forces and pressures were found on “sternum right”, with the highest values recorded with harness 1. The average peak force (303 kPa [30.3 N/ cm^2^]) was comparable to what has been observed in horses ridden with a bit [42], but the authors noted that when accounting for size, the load exerted on the trunk of guide dogs was higher in relation to their body mass compared to that of horses. While the regions “sternum right” and “sternum left” were almost constantly loaded, all other regions exhibited smaller forces and pressures. The authors highlighted that, contrary to previous assumptions, the back regions were not heavily loaded, which could be due to the lifting of the handle. They also suggested that the difference between the right and left sternum pressure may be due to the person walking on the right side of the dog while keeping a constant tension on the harness using the handle.

An abstract from Galla et al. [68] examined the influence of the harnesses on spine movement on what appears to be the same sample of dogs (eight qualified guide dogs) while walking straight as well as turning to either side. Harness 1 restricted latero-lateral movement compared to when dogs walked without a harness, in all three directions. Additionally, harnesses 1 and 3 restricted the dorso-ventral movement when turning right compared to walking straight. The authors concluded that at least one harness (harness 1) was found to influence the kinematic movement of guide dogs.

Knights and Williams [39] evaluated the influence of three types of working harnesses on thoracic limb stride length as well as room of movement of the shoulder, elbow, and carpal joints. The harnesses were two types of Y-shaped harnesses and the usual working harness the dog used, while a standard leather collar was used as a control. Harness 1 had a B-type handle (triangular shape handle that fits more laterally around the chest strap), while harness 2 and the original harness of the dog had an A handle (rectangular handle that fits more upright onto the dorsal part of the harness). They tested 13 Labrador, Golden, or crosses of Labrador and Golden Retrievers in training to be assistance dogs. Dogs were recorded walking in a straight line on a walkway for 2 min. Each dog was tested using all four restraint devices across three trials, following a randomized Latin Square design. Kinematic analysis software tracked tape markers placed on the left side of the dog on key areas of the forelimbs. The results indicated that wearing a harness influenced thoracic limb kinematics, especially in the case of the harness with a B-type handle which resulted in the most significant restriction to stride length and reduction in joint room of movement. The authors suggested that this type of handle may increase proprioceptive input from the harness, having a greater impact on the dog. The authors noted that B-type harnesses are commonly used for handlers who need to better perceive the dog’s movement. More research is needed to explore the impact of these types of handles, particularly for dogs who are expected to walk daily in a harness.

Weissenbacher et al. [34] evaluated the impact of two types of harnesses on the force distribution between the paws of 12 certified guide dogs. The dogs were examined walking with a collar and leash, walking with their usual harness (chest-strap with straight handle), and walking with a Y-shaped harness, with either a straight or a curved handle. Ground reaction forces were measured using a pressure plate integrated into the floor. Dogs were led back and forth several times until they showed a smooth gait pattern, and then measurement was started. All dogs were led on the left side by the same sighted handler. The order of measurements was randomized. There were no differences in the comparison of walking with a collar versus walking with either type of harness when using a leash.

Conversely, both harnesses had an effect on ground reaction forces and stride length when dogs were led using a handle, but contrary to the authors’ expectations, there was no evidence of a curved handle having less impact than a straight one. For both harnesses, regardless of handle type, there was an increased impulse in the right hind limb, which was compensated by a decreased impulse in the left forelimb. The Y-shaped harness showed an additional impact on shortening stride length. The authors concluded that wearing the Y-shaped harness with a handle had a greater effect on the dog’s biomechanics than a chest-strap harness.

Sandberg et al. [40] examined the impact of wearing a tactical harness on the motion of the thoracic limbs. They tested five adult mixed-breed dogs wearing a custom-fit tactical harness. Reflective markers were used to determine three-dimensional dynamic motion and establish movement for the shoulder, elbow, and carpus using motion capture data when the dog was with and without the harness. Wearing the harness affected measures of the carpus and the elbow at both walk and trot, while effects on the shoulder were only observed at a walk.

### 3.6. Studies Focusing on Leash Tension

See Supplementary Table S6 for detailed results of studies exploring the effects of the tension of the leash itself, regardless of the restraint device worn by the dog.

Shih et al. [54] set out to validate a canine leash tension metre that recorded tension and direction, differentiating if the pulling was initiated by the dog or the handler, in order to examine dog–human interactions during walks. They considered the effects of age, size/weight, and in-shelter behaviour in 111 shelter dogs. Shelter staff had already categorized dogs into walking levels according to how well behaved they typically were during walks (level 1 dogs walked on a loose leash most of the time, level 2 dogs pulled occasionally, and level 3 dogs tended to pull). All dogs wore both a collar and a front-clip harness, and the leash connected with a tension metre was clipped to both at the front of the dog’s chest. Larger and heavier dogs exerted higher leash tension but pulled less frequently than smaller ones, and this was mirrored by the behaviour of the human. In the same line, younger dogs pulled more frequently, and handlers reacted by pulling on the leash more often when walking them. Dogs that staff considered well-behaved during walks created lower leash tension, but handlers did not respond with lower forces, as handler behaviour did not differ based on walking level. The authors concluded that the canine leash tension metre provided a robust approach to explore real-world walking scenarios.

Van Hernwijnen et al. [69] assessed leash tension in dog–human dyads walking on courses with distractions. The first course consisted of a 12 m straight path with pieces of chicken placed at fixed positions on either side of the path. The second course was considered more difficult, as it was a zigzag path of 12 m with objects such as balls, fake dogs, food bowls, and oddly shaped items placed at the sides. They tested 24 dogs who walked with their guardians while wearing their usual restraint device (either a flat collar or standard harness). The results indicate that leash tension was 1.6 times higher in the second course, in line with the authors’ prediction that tension would increase in the most challenging course. Moreover, the tension during both courses was correlated within a dyad, suggesting consistent differences between dyads. The authors concluded that further research is needed to establish how much of the tension can be attributed to the dog compared to the guardian, and to further explore how leash tension characterizes dog-guardian interactions.

## 4. Discussion

It is important to note that there is currently a lack of epidemiological data and clinical case studies about actual injuries associated with the use of restraint devices in dogs. Because of this, the reviewed studies focus on the potential for injury to base their welfare concerns. For example, if recorded pressures exceed known thresholds for tissue damage, it could be reasonable to infer a risk, even in the absence of reported clinical cases. However, this absence of evidence may also reflect underreporting or limited surveillance rather than an absence of harm, as some injuries may not be explicitly linked to the use of restraint devices in clinical records.

A careful analysis of the literature suggests there is no restraint device that can be universally recommended. However, some general conclusions can be drawn despite the lack of a clearly optimal solution applicable to all dogs. Table 1 outlines some of the general benefits and drawbacks of each device, although it should also be noted that there may be multiple ways of using the same equipment, and considerations about tightness and overall fit are essential.

For flat collars, the key findings were that pulling while wearing a collar was found to increase intraocular pressure [46,47] and could cause damage to the neck [64]. Collars were found to exert pressures which were higher than ill-fitting saddles on horses [45,62], and ranged upward from 83 kPa, when values such as 4.3 kPa and 11 kPa have been indicated as thresholds for potential injury in humans and horses, respectively [45]. Additionally, Bailey et al. [46] found that simply wearing a collar increased intraocular pressure in brachycephalic and dolichocephalic dogs when walking even on a loose leash. Moreover, in the case of brachycephalic dogs, this effect was also observed while they were stationary, and wearing a collar also increased their respiratory rate. These findings support the recommendation that neck collars should not be used on brachycephalic dogs.

Bailey et al. [43] highlighted welfare concerns associated with collar use, as they observed that some dogs wearing a collar pulled with force exceeding 80% of their body weight. Taking these findings together, guardians should explore alternative restraint methods in the case of dogs that pull and/or have conditions where an increase in intraocular pressure could be harmful [46,47,64].

Moreover, Hunter et al. [45] indicated that the direction in which the dog walked affected the amount of pressure applied to the neck, which was higher when walking in circles compared to walking in a straight line. In the same line, van Herwijnen et al. [69] reported increased leash tension when walking on a complex zigzag course compared to a straightforward one, both when wearing a harness or a collar. Taking this into account, particular vigilance should be exercised when using collars in situations where tighter control is needed (e.g., navigating busy sidewalks). Finally, in terms of the construction of the collar, both Carter et al. [64] and Hunter et al. [45] found that padding appeared to have a paradoxical effect by reducing the area of contact and thus concentrating pressure on a smaller area. This is a noteworthy finding, as it challenges the logical assumption that a padded collar would provide more cushioning and be less likely to cause injuries to the neck. While evidence is not yet conclusive enough to discourage the use of padded collars, this is an interesting finding that should be explored in more detail, and guardians should consider the amount and distribution of padding when selecting a collar.

For both collars and harnesses, generally, there were no differences in behavioural stress signs when wearing a harness or a collar [57,59], or the differences were minimal and inconclusive. Additionally, Zilocchi and Parisi [67] reported there were no differences in terms of dog-dog communication when wearing either of these devices. Regarding human-directed behaviour, one study reported that dogs looked more frequently at the experimenter when wearing a harness in a condition in which there was food [59], and another indicated a trend in the same direction with a higher frequency of looking at the handler while wearing a harness [58]. However, a different study found opposite results as dogs exhibited more attention-seeking behaviours towards the guardian when wearing a collar [67], and in another one, there were no differences in human-directed behaviour according to the restraint device [57]. It is possible that these discrepancies are related to subtle differences in methodology and settings. Zilocchi and Parisi [67] proposed that dogs may experience greater difficulties in intraspecific communication while wearing a collar and may have turned to their guardian for guidance. However, the theoretical basis for this explanation is unclear, and they acknowledged that further research is needed to clarify the underlying mechanisms. Shih et al. [59] suggested that dogs may have directed their gaze toward the experimenter due to the presence of food and proposed that this behaviour may have occurred more frequently with the harness because it was more comfortable and less restrictive. In sum, the current evidence does not show a difference between collars and harnesses in causing stress-related behaviours in dogs.

Regarding pulling behaviour, both Shih et al. [59] and Bailey et al. [43] reported that dogs pulled more when wearing a harness clipped at the back compared to a collar. This suggests that the pressure of collars is uncomfortable enough to reduce the behaviour of pulling as compared to the pressure of harnesses.

Moreover, Bailey et al. [43] found that smaller dogs exhibited higher mean pulling forces than medium or large dogs, reaching as high as 122% of their body weight. This is interesting as the average peak force observed on the sternum of guide dogs using harnesses [12] was similar to the pressure measured on the neck when wearing a collar [45]. This was in line with prior observations of dogs pulling wheelchairs or sleds [63], as well as horses ridden with a bit [44]. However, as noted by Peham et al. [12], when adjusted for size, dogs carried a heavier load relative to their body mass than horses. Conversely, Shih et al. [54] reported that larger and heavier dogs exerted higher leash tension but pulled less frequently than smaller ones, and this was mirrored by the behaviour of the human holding the leash. Bailey et al. [43] indicated the reason for this was unclear, as previous research had been in line with Shih et al. [54] and found that larger dogs typically exhibited increased parameters, but in one case, these differences were reduced when normalizing for body weight [70]. They suggested larger dogs may have received more training and be more obedient (and pull less) as a result.

Harnesses clearly influenced canine gait and restricted the movement of the shoulder, while some restricted the elbow as well [32,38,40,65,66]. In addition, Lafuente et al. [32] found that harnesses that are typically considered “restrictive” (i.e., chest strap harnesses) ended up limiting the movement of the limbs less than other harnesses that were deemed “non-restrictive” such as Y-shaped ones. Similarly, Weissenbacher et al. [34] concluded that wearing the Y-shaped harness with a handle had a greater effect on kinematics parameters in guide dogs than wearing a chest-strap harness with a handle. These results are interesting, as the common perceptions and assumptions about these restraining devices have not been supported in the studies. Nevertheless, as noted by Wiliams et al. [40], it is possible that the amount of restriction the horizontal strip causes to the movement of the thoracic limbs depends on the specific anatomy of the dog, being more or less restrictive depending on where it sits in relation to the elbows and the shoulders.

For other parameters, the differences across harnesses were harder to categorize. Pálya et al. [65] indicated that both chest strap and Y-shaped harnesses altered gait parameters, without one being clearly superior to the others. In the same line, Wiliams et al. [38] found that there were differences between these types of harnesses in body weight distribution, stride length, and humerus angulation, but they were not consistent across dog breeds or harness designs. Sandberg et al. [40] also reported that wearing a tactical harness (details not further specified) affected the gait of all joints of the forelimbs. Conversely, Kiss et al. [37] did not find differences in gait patterns across chest-strap and Y-shaped harnesses, and Weissenbacher et al. [34] found no differences comparing two types of harnesses and a collar when walking on leash (they did find differences when they were worn with special handles as noted above). Dowdesdwell & Churchill [66] concluded, recommending the use of chest-strap or H-shaped harnesses, as they found these were the ones that affected fewer kinematics parameters. These findings suggest that while all harnesses will affect a dog’s gait, chest-strap and Y-shaped harnesses are likely to be the least restrictive.

Turning to studies on working dogs’ harnesses, differences in stride length and joint range of motion were observed in guide dogs wearing harnesses with handles compared to collars [39]. Effects on ground reaction forces and stride length were also reported when dogs wore a harness attached to a handle [34]. Some differences were observed in terms of the specific designs of the handles, but more research is needed to identify the least disruptive style, as handles are required for guide dogs to perform their duties. Some areas to explore include the use of single bar handles [39], as well as the effects of the way the handle is attached to the harness, such as the angle of attachment and the type of connector used [34].

In terms of the location of the clip when harnesses are used with a traditional leash, Dowdesdwell & Churchill [66] observed that use of front-clip harnesses was related to the largest impact on joint flexion and extension. In line with this, Johnson & Wynne [58] indicated that in their study, dogs pulled more when wearing a martingale collar than a harness, but in their case, the harness was connected at the front which may create higher discomfort when pulling.

Moving on to other types of restraint methods, head collars are devices worn over the muzzle that clip at the back of the head or the chin. This typically results in the dog’s head being turned to the side when they pull, and manufacturers recommend using the back clip for better control [61]. Ogburn et al. [60] suggested head collars work because dogs respond by pulling back from the pressure concentrated on the back of the neck, instead of pulling forward as they would do with other types of restraint. Some authors have indicated these collars allegedly mimic what they called “natural dominance behaviours”, increasing “deference” and “obedience” towards the handler [60,61]. However, the dog’s apparent increase in obedience while wearing this device may also be due to overall suppression of behaviour [61], likely due to its aversive nature.

In line with their predictions, Ogburn et al. [60] found that dogs wearing a head collar behaved in a more controlled way during obedience exercises and required less repositioning during the measurement of physiological parameters of stress. However, while there were no significant differences in those physiological parameters when wearing a head collar compared to a traditional collar, dogs showed behavioural stress signs such as crouching and lowered heads and ears when wearing the head collar. Moreover, they also pawed at their noses and fought the leash, which suggests they experienced some discomfort wearing this device. Haug et al. [61] compared four types of head collars and found no differences in stress signs while using them, but it should be noted that they did not include a control condition in which dogs wore another type of restraint device such as a traditional collar. Unfortunately, the authors only reported group comparisons and did not include the amount of collar-directed and walking refusal behaviours that occurred. However, visual inspection of their figures suggests both of these groups of behaviours were present for all collars, in particular during the first session. They suggested dogs adapted quickly to the collars as there was a decrease in these behaviours across sessions, but it should be taken into account that some of these were still present in the final one. Moreover, this study included a phase in which the head collar was unclipped from the leash to test whether dogs would be able to remove it. One dog managed to do so (and there is no report on how many others attempted it), which again suggests that at least one dog out of twelve experienced discomfort wearing this device.

As Dodgewell and Churchill [66] noted, it is not possible to identify a single ‘best’ device; instead, the goal is to provide information that helps guardians to make informed decisions based on the specific needs of their dog and situation. Guardians should carefully weigh the potential benefits and drawbacks of each option in relation to their circumstances. Some of the factors to take into account include the anatomy of their dog (e.g., where the device sits on their body, head shape), the characteristics of their typical walks (e.g., type of terrain, amount of tight control needed), and the functionality required from the device (i.e., whether no-pulling features are needed).

For instance, considering that not walking the dog due to a lack of control can also negatively impact their welfare, and letting the dog off-leash is not feasible in all cases, guardians concerned about pulling may consider using “no-pulling” restraint devices. In this case, guardians should be aware that all “no-pulling” devices ultimately work by creating discomfort when pulling in order to discourage the pulling behaviour. More research is needed to better characterize the potentially aversive nature of restraint devices that are not typically considered as such but, nonetheless, work in a manner that creates discomfort by tightening (martingales, some harnesses) or creating pressure that leads to redirection when pulling (head collars, front-clip harnesses). Ultimately, guardians must weigh the functional benefits of improved control against the possible welfare costs of employing such devices. Williams et al. [38] also noted that chest-strap harnesses, which may be less restrictive to some dogs due to the particularities of their anatomy, may also be less effective in discouraging pulling, further highlighting the need to consider trade-offs of discomfort to the dog and pulling on the leash.

Based on the benefits and drawbacks outlined in Table 1, when pulling is a concern, it may be appropriate to consider restraint devices intended to reduce pulling in decreasing order of invasiveness. Front-clip harnesses that do not tighten seem generally preferable to harnesses that tighten, followed by martingale collars (when fitted to tighten beyond that of a flat collar) and head collars. Front-clip harnesses are typically designed to reduce pulling by introducing slight discomfort when the dog pulls and redirecting the direction of movement. This appears to be a more humane option for dogs that are otherwise difficult to manage on leash (especially when guardian strength or balance is a concern) than devices that tighten, or head collars, which also work through redirection but have been associated with increased stress. However, it is important to recognize that any device used to inhibit pulling is likely to introduce some degree of aversiveness to the walk. This highlights the importance of using humane, reward-based training techniques to address pulling behaviour, minimizing the need for devices that prevent it through tightening or redirection.

In the case of dogs that do not pull, the drawbacks of using equipment designed to prevent pulling generally outweigh the potential benefits. For these dogs, the most suitable option may be back-clip harnesses, especially chest-strap and Y-shaped models. However, as back-clip harnesses have been shown to affect gait parameters in some studies, dogs that consistently walk on a loose leash and do not require tight control may benefit from using a traditional flat collar instead. Care must be taken that any padding does not actually concentrate force over a smaller area. For dogs whose head and neck circumference are similar (e.g., sighthounds) but who do not typically pull, the use of martingale collars may be an option to reduce the likelihood of escape through backing out.

Besides selecting an appropriate restraint device, ensuring a proper fit is equally important. An ill-fitting device can place pressure on unexpected areas of the body or excessively restrict movement, as it has been observed in horses wearing poorly fitted saddles [62]. Moreover, Haugh et al. [61] indicated that ill-fitting head collars may restrict the opening of the mouth and apply pressure on the lips, which could increase discomfort and affect the dog’s tolerance to the device. In the case of harnesses, some of the recommendations for the best fit include being able to fit one or two fingers between the harness and the body. This helps ensure the harness is not too tight, while also preventing it from being so loose that it causes chafing or allows the dog to slip out of it [30]. For some equipment, like martingale collars, fit will fundamentally alter the function: when fitted tightly, a martingale will function like a choke collar. Furthermore, a poor (i.e., loose) fit may result in the dog being able to slip out of the device. Townsend et al. [6] suggested that veterinarians should inform guardians on how to fit these devices which would increase compliance, satisfaction, and welfare while reducing the risk of injuries. This recommendation may be extended to other canine professionals who regularly interact with guardians and their dogs, such as trainers and groomers. In addition, reward-based training protocols can help dogs become more comfortable wearing restraint devices [71,72].

Future research should explore the impact of specific factors such as padding, strap width, and material type in more detail [38,46]. Moreover, not enough attention has been given to the previous history of using specific devices [57], as well as to possible longitudinal effects of long-term use. Additionally, Blake et al. [73] pointed out that the use of restrictive restraint devices to prevent pulling may be more frequent when dogs are young and are undergoing training to walk on a loose leash. They highlighted that this may have a greater impact on the musculoskeletal systems and their developing growth plates than using these devices on adult dogs. Considering this, research could also explore the impacts of different types of restraint devices at different ages. Another potential focus for future studies relates to the role of the handler’s behaviour in leash pulling. For instance, individual differences in leash tension were observed across dyads of guardians and their dogs [69], and handlers did not always respond with lower forces holding the leash when walking well-behaved dogs that did not pull [54]. Taken together, these findings suggest there may be human factors underlying leash tension, highlighting the importance of further research into handler-related influences on dog pulling behaviour.

Another important area to explore relates to training, both in terms of techniques to help reluctant dogs become comfortable with wearing restraint devices, as well as the best protocols to humanely teach dogs to walk without pulling to minimize the need for no-pulling equipment. Additionally, individual dog factors should be explored in more detail, both in terms of their anatomy as well as their life experiences and behaviour, including aspects like their level of training and overall personality [43]. Finally, it remains to be seen how dogs perceive various restraint devices and whether, if given a choice, they would show a preference for one device over another. Understanding individual preferences is likely to decrease the aversive nature of restraint devices as well as having the potential to introduce more agency to the dogs’ lives, which has been suggested to improve welfare outcomes [74].

## 5. Conclusions

Selecting and fitting restraint devices for dog walking requires careful consideration of both functional needs and individual characteristics. The choice of restraint device should depend on how much the dog pulls and how this affects their guardian’s ability to walk them safely. Due to potentially harmful physical effects, flat collars should not be used on brachycephalic dogs. For dogs that pull, besides training, the recommendation is to first consider non-tightening front-clip harnesses, as they offer better control without being as restrictive as tightening harnesses, martingale collars, and head collars, which should be considered as a last resort. For dogs that do not pull, chest-strap and Y-shaped harnesses clipped at the back appear to be the most suitable, while collars may be appropriate for dogs that routinely walk on a loose leash.

## Figures and Tables

**Table 1:** Outline of benefits and drawbacks/considerations for the restraint devices considered in this review.

Device	Benefits	Drawbacks/Considerations
Collar	Does not alter gait parameters Does not restrict movement of the joints and limbs Is not associated with increased signs of stress compared to other devices	Wearing a collar while walking is associated with increased intraocular pressure and pressure on the neck for dolichocephalic and brachycephalic dogs Increased respiratory rate observed in brachycephalic dogs simply wearing collar Padding may create uneven pressure May allow dogs with similar circumference of the neck and head to escape
Martingale collar	Comparable to a flat collar when fitted to avoid excessive tightening Prevents escape for dogs with similar circumference of the neck and head.	Comparable to a choke collar when fitted to allow excessive tightening
Head collar	Does not alter gait parameters Does not restrict movement of the joints and limbs Reduces pulling	Associated with behavioural signs of stress, removal attempts, and refusal to walk May cause increased pressure on the muzzle or the nose. Some models were associated with an increased risk of the dog slipping out of the device
Harness back clip	Does not increase pressure on the neck or intraocular pressure Is not associated with increased signs of stress compared to other devices	Can alter gait parameters Can restrict movement of the joints and the limbs Padding and strap width may create uneven pressure Associated with increased pulling Chest-strap and Y-shaped harnesses are likely to be the least restrictive
Harness front clip	Does not increase pressure on the neck or intraocular pressure Reduces pulling	Can alter gait parameters Can restrict movement of the joints and the limbs Potential for discomfort, especially in models that tighten Chest-strap and Y-shaped harnesses are likely to be the least restrictive

## Data Availability

No new data were created or analyzed in this study. Data sharing is not applicable to this article.

## Supplementary Materials

The following supporting information can be downloaded at: https://www.mdpi.com/article/10.3390/ani15152162/s1. Table S1: Studies comparing collars, Table S2: Studies primarily comparing harnesses, Table S3: Studies comparing harnesses and collars, Table S4: Studies comparing head collars, Table S5: Studies comparing specialized equipment in working dogs, Supplementary Table S6: Studies focusing on leash tension. Supplementary Table S7: Evidence-based findings on effects of different types of restraint equipment for walking dogs.
