# Supplementary material for: Review of Collars, Harnesses, and Head Collars for Walking Dogs
Source: Animals (Basel). 2025 Jul 22;15(15):2162. doi: 10.3390/ani15152162 (PMC12345489; doi:10.3390/ani15152162)
Supplement: Supplementary file 1 [file animals-15-02162-s001.zip › animals-3767813-supplementary.pdf]

**Supplementary Table S1.** Studies comparing collars

| | Sample | Type of restraints used | Design | Measurements | Key findings |
|---|---|---|---|---|---|
| Carter et al. [64] | PVC neck model (no dogs included in the study) | Seven collars and a slip lead:<br>- Padded webbing collar: Wainwrights (model not specified)<br>- Rolled collar: 3 peaks (model not specified)<br>- Padded sports collar: Non Stop (Polar)<br>- Lurcher collar: Pampeano (model not specified)<br>- Leather and thread collar: Pampeano (Navidad)<br>- Flat webbing collar: Pets at home (model not specified)<br>- Rope slip-lead: Brand and model not specified<br>- Choke chain: Ancol Heritage (Extra Heavy Check Chain)<br><br>Picture: Yes | A range of forces were applied to the leash representing different interactions: firm pull (40 N), strong pull (70 N) and jerk (mean force 141 N) | Contact area of the collar and pressure on the neck were measured with a pressure sensor on the neck model | - All collars had the potential to cause injury when pulling<br>- Padding may concentrate pressure in a smaller surface area |
| Hunter et al. [45] | N = 8<br>Population = Pets<br>Weight: Mean 31.4 ± 11.40 kg (13.2 - 55 kg)<br>Breed: Various<br>Age: Mean age was 6.5 ± 3.38 (2 – 12)<br>Sex: Not reported | Three types of collar:<br>- Double-layer polyester and nylon weave: Great and Small (model not specified)<br>- Single layer nylon weave: Great and Small (model not specified)<br>- Single layer canvas: Great and Small (model not specified)<br><br>Picture: Yes | Walking path with 3 segments followed in order: clockwise, counterclockwise, straight<br>Repeated 3 times<br><br>Order: randomized<br><br>Leash length kept the same for all but not specified<br><br>Handler identity: Same for all<br>Handler side: Left | Peak force and contact pressure measured with a commercially available device (F-Scan In-Shoe Analysis Sensor) placed ventrally between the collar and the neck of the dog | - Collar material altered contact pressure and peak force. These were higher under the double layer polyester and nylon padded collar<br>- Direction of travel also affected force, being highest in a counter-clockwise direction, and lowest in a straight line |

**Supplementary Table S2.** Studies primarily comparing harnesses

| | Sample | Type of restraints used | Design | Measurements | Key findings |
|---|---|---|---|---|---|
| Dowdeswell & Churchill [66] | N: 30<br>Population: Pets<br>Weight: 16 – 36 kg<br>Breed: Not reported<br>Age: > 1.5 years<br>Sex: Not reported | 6 types of harnesses:<br>- Chest strap ("straight front"), Julius K9 "IDC Powerharness".<br>- Y-shaped/clipped at the front ("front-clip"): Ruffwear "Front Range Dog Harness" | Walkway of 4 m, repeated 3 times per device.<br><br>Order: randomized<br><br>Leash length: 1.3m. | The gait cycle was broken into elbow extension, elbow flexion, shoulder extension and shoulder flexion. Videos were analysed with | - Elbow extension was most affected by the Y-shaped harness clipped at the front as well as the no-pull harness; and not |

| | | | | | |
|---|---|---|---|---|---|
| | | - Chest-plate: EzyDog "Chest Plate Dog Harness"<br>- No-pull: Halti "No pull harness:<br>-Step-in: 3 peaks "Step-in harness".<br>- H-shaped ("Y-front"): Halti "Walking harness"<br>- Collar: Halti "Comfort collar:<br><br>Picture: Yes | Clipped at the back except Ruffwear Front Range harness which is referred as "front-clip".<br><br>Handler identity: Owner<br>Handler side: Not reported (but supplementary pictures show dogs walking on either side) | Quintic Biomechanics Software and Quintic Manual Angular Software | affected by the chest-strap or H-shaped harness<br>- Elbow flexion was most affected by the Y-shaped harness clipped at the front and step-in harnesses, and not affected by the chest-strap or H-shaped harness<br>- Shoulder extension was affected by all harnesses compared to the collar<br>- Shoulder flexion was most affected by the chest-plate, Y-shaped clipped at the front, step-in and H-shaped harness, and not affected by the chest-strap harness<br>- Based on results authors do not recommend front-clip Y-shape harnesses, and instead prefer the chest-strap or H-shaped ones |
| Lafuente et al. [32] | N: 9<br>Population: Pets<br>Weight: Not reported<br>Breed: Various<br>Age: > 1 year old<br>Sex: Not reported | 2 types of harnesses:<br>- Y-shaped ("non-restrictive"): Trixie "Fusion Harness"<br>- Chest strap ("restrictive"): PetSafe "Easy walk nylon harness"<br><br>Picture: Yes | Walking and trotting on canine treadmill (Starkerhund SM01), comfortable speed determined for each dog during habituation<br><br>Order: fixed (no harness, Y-shaped, Y-shaped with 2.5 barbell weights clipped, chest strap, chest strap with added weights) | Shoulder extension measured with markers placed on the left side of the dog | - Harnesses limit shoulder extension<br>- Harnesses typically considered "non-restrictive" may be more restrictive in some cases |

| | | | | | |
|---|---|---|---|---|---|
| | | | Leash clipped at the back, length not specified<br><br>Handler: N/A | | |
| Kiss et al. [37]<br><br>This is a research report (i.e., not peer reviewed) available on Julius K9 website | N: 5<br>Population: Pets<br>Weight: 3 – 50 kg (mean not provided by the authors, calculated for this review based on available raw data = 24.2 kg)<br>Breed: 2 Bullterrier, 1 Cane Corso, 1 Yorkshire Terrier, 1 Beagle-Labrador mix<br>Age: Not reported<br>Sex: Not reported | 3 harnesses:<br>- Julius K9 “K9”<br>- Julius K9 “Duo-Flex”<br>- Julius K9 “IDC”<br>- No harness<br><br>Characteristics of each harness are not specified, but see below Pálya et al. (2022)<br><br>Picture: No | Walking on ground and treadmill (brand not specified)<br><br>Order: not mentioned<br><br>Retractable leash attached at the back<br><br>Handler identity: N/A<br>Handler side: N/A. On leashed trials a retractable leash was held behind the dog. The owner encouraged the dog to move by squatting in front of the treadmill or calling from across the room | Walking kinematics (step width, step height, step length, stride length, spinal and limbic joint angle) measured with OptiTrack Flex13 motion capture system using infra reflexive markers<br><br>Force of the leash was measured using a load cell | - Walking surface (ground vs treadmill) and the presence of a leash affected movement<br>- However, no significant differences in gait patterns were noted across harnesses with no leash.<br>- The IDC harness showed a small but significant reduction on pelvic limb step width on the treadmill with a leash, while there were no effects on step/stride length |
| Pálya et al. [65] | Note these appear to be the same dogs as in Kiss et al. (2018) with the exclusion of the Cane Corso.<br><br>N: 4<br>Population: Pets<br>Weight: 3 – 26 kg (mean not provided by the authors, calculated for this review based on available raw data - all weights: 3, 16, 26, 26 – mean = 17.7kg)<br>Breed: 2 Bullterrier, 1 Yorkshire Terrier, 1 Beagle-Labrador mix<br>Age: 3-5 years<br>Sex: Not reported | 3 harnesses:<br>- Chest strap: Julius K9 “K9 Power Harness”<br>- Chest strap: Julius K9 “IDC”<br>- Y-shape: Julius K9 “Duo-Flex”<br>- Y- shape: Fressnap (model not specified). Note this harness was only used for 1 dog and it is not included in all analyses.<br>-No harness<br><br>Picture: yes | Treadmill ProFitness L150 treadmill.<br><br>Order: Fixed (no harness, each harness with no leash, each harness with leash).<br><br>Other procedures same as in Kiss et al. (2018) | Spatio-temporal gait parameters, paw movement, limb joint angles and spinal angles measured with OptiTrack Flex13 motion capture system using infra reflexive markers | - All harnesses altered some gait parameters, without one being clearly superior to the others<br>- Leashed walks differed the most from the baseline (walking with no harness)<br>- Y-shape harness reduced shoulder room of motion for 2 out 3 dogs, especially when leashed |
| Wiliams et al. [38] | N: 66 | 3 harnesses | Dogs were filmed for one motion cycle, | Stride length, body weight | - There were differences in |

| | | | | | |
|---|---|---|---|---|---|
| | Population: 40 pets, 26 prison detection dogs<br>Weight: Mean 18.99 ± 7.6 kg.<br>Breed: 28 Cocker Spaniels, 14 Labrador Retriever, 6 Springer Spaniels, 6 Staffordshire Bull Terriers, 4 French Bulldogs, 9 mixed breeds.<br>Age: 1-10 years<br>Sex: 41 male, 25 female | - Y-shape: Ruffwear (model not specified).<br>- Y-shape: Perfect Fit (model not specified).<br>- Chest-strap: Julius K9 (model not specified).<br><br>- Collar only (brand and model not specified)<br>- Own harness the dog typically used a different one<br><br>Picture: yes | defined as the retraction of the limb during the stance phase and the protraction during the forward swing phase<br><br>Order: randomized<br><br>Leash attached at the back, length not specified but it was kept loose (trial was repeated if dog pulled or left the mat)<br><br>Handler identity: Owner<br>Handler side: Not reported (pictures show dogs walking on either side) | distribution across the front versus the back paws, angulation of the humerus while in motion. Measured using a pressure sensing mat (Tekscan Strideway gait analysis system) as well as video footage | body weight distribution, stride length and humerus angulation but they were not consistent across dog breeds or harness designs<br>- Chest-strap harness restricted elbow movement in Cocker and Springer Spaniels and shoulder movement compared in Labradors, Staffordshire Bull Terriers and mixed breed dogs<br>- Chest-strap harness affected stride length in French bulldogs, Cocker Spaniels and mixed breed dogs<br>- Y-shaped harness affected stride length in Labradors and Springer Spaniels |

**Supplementary Table S3.** Studies comparing harnesses and collars

| | Sample | Type of restraints used | Design | Measurements | Key findings |
|---|---|---|---|---|---|
| Bailey et al. [43] | N: 28<br>Population: Pets<br>Weight: Grouped as small (n = 8, Mean 7.5 ± 1.3 kg), medium (n = 8, Mean 21.5 ± 2 kg) and large (n = 12, Mean 31 ± 2.2 kg)<br>Breed: 8 Mixes, 2 Norwich Terriers, 2 Siberian Huskies, and 1 Maltese, Dachshund, Shiba Inu, Dalmatian, Australian Cattle Dog, Labrador | - Y-shape: Good2Go (model not specified).<br>- Collar: Tactipup (flat nylon, model not specified)<br><br>Picture: yes | Each dog completed a 15m~ circuit twice (once with the collar and once with the harness). The circuit included an unknown dog, food and a thrown toy<br><br>Order: Randomized<br><br>6ft leather leash. Held hands free (connected at the waist and tights with a climbing harness). | Peak force, mean force, and leash tension time in a single pulling event, measured with a digital dynamometer (Meloq EasyForce®) affixed between the collar or harness and the leash for each dog | - Dogs pulled more when wearing a harness compared to a collar<br>- Smaller dogs pulled more |

| | | | | | |
|---|---|---|---|---|---|
| | Retriever, Australian Shepherd, American Pitbull Terrier, Labradoodle, Cane Corso, Dobermann, Newfoundland, Great Dane, Border Collie, Belgian Malinois, and German Shepherd.<br>Age: 1 – 11 years<br>Sex: Not reported | | Harness was clipped at the back<br><br>Handler identity: Researcher (not mentioned if it was the same for all).<br>Handler side: Not reported | | |
| Bailey et al. [46] | N: 20<br>- 10 brachycephalic<br>- 10 dolichocephalic<br><br>Population: Pets<br>Weight: mean body condition score (BCS) of 6.6 ± 1.23 (range 5–8)<br>Breed: Various<br>Age: 4.5 ± 2.97 years (8 months – 8 years)<br>Sex: 12 males (8 neutered), 8 females (1 spayed) | - Collar: Ruffwear (Front range), width 2-2.5 cm<br><br>- Y-shaped: Ruffwear (High and light) | Five conditions: baseline, collar or harness while stationary (leash was kept taut for 10 s while the dog stood), collar or harness with exercise (dogs were walked on a loose leash back and forth along an enclosed 43m grass area. The handler tried to keep walking speed consistent). Intraocular pressure and respiratory rate were measured immediately afterwards in each condition.<br><br>Order: randomized (except for baseline which was first). 10 min break between conditions<br><br>Harnesses were clipped at the back<br><br>1 m leash<br><br>Handler identity: Researcher (same for all), side not specified | Intraocular pressure measured by rebound tonometry using an iCare TonoVet tonometer<br><br>Respiratory Rate was considered as breaths per minute and measured visually and/or through contact examination for 15s then multiplied by 4 | Intraocular pressure:<br>- Increased in brachycephalic dogs wearing a collar while stationary, but not in dolichocephalic dogs<br>- Increased in both groups wearing a collar while exercising<br>- Not increased in either group when wearing a harness<br><br>Respiratory rate:<br>- Increased in brachycephalic dogs while stationary with a collar, and while exercising with either a collar or a harness, with no differences between devices<br>- No effects observed in dolichocephalic dogs |
| Grainger et al. [57] | N: 30<br>Population: Pets<br>Weight: Not reported<br>Breed: 50% purebred, 50% | Harness:<br>- Y-shape: Dog Games Ltd "Perfect Fit Harness".<br><br>Collar: | 20 min walk (only the middle 10 min recorded and analyzed).<br><br>Order: First visit using the restraint | Focal sampling of stress behaviours (licking lips, yawning, low body position, low tail position, ears low or pulled | - Low frequency of stress behaviours<br>- No differences between collar and harness<br>- Dogs with a history of being |

| | | | | | |
|---|---|---|---|---|---|
| | mixes. Not further specified.<br>Age: > 18 months.<br>Sex: 19 males (14 neutered), 11 females (9 neutered)<br><br>Prior history: 15 dogs had previous experience being walked with collars and 15 with this harness | - Dog Games Ltd (fleece lined collar) – for dogs who did not have their own.<br>- Collars "from various manufacturers" when dogs brought their own collar.<br><br>Picture: yes | they were used to wearing. Second visit after a week of habituation to the other restraint.<br><br>1 m leash. Not specified if attached at the front or the back.<br><br>Handler identity: Owner<br>Handler side: Not reported<br>Handler behaviour: Instructed to maintain walking space and avoid communicating with the dog | back, vocalizations, paw lifting, looking at owner, panting, and trembling/body shaking) and behaviours related to potential restriction of movement (sniffing ground, tracking, and stopping) | walked on a collar showed increased low ear position |
| Johnson & Wynne [58] | N: 23<br>Population: Shelter<br>Weight: 13-42 kg (mean not provided by the authors, calculated for this review based on available raw data. Mean = 29.56 ± 7.06)<br>Breed: 19 mixes, 3 German Shepherds, 1. Doberman.<br>Age: 1- 10 years (estimated)<br>Sex: Not reported | - Martingale 1" fabric collar: Radio Systems Corporation<br>- Front clip: 2 Hounds Design (model not specified)<br><br>(Note the authors also tested two types of prong collars, not included in this review).<br><br>Picture: yes | 5 min walk<br><br>Order: Fixed (martingale collar, front-connection harness). 2 hr break in home kennel between walks.<br><br>1.52 m nylon leash<br><br>Handler identity: Researcher (not reported if it was the same for all)<br>Handler side: Not reported<br>Handler behaviour: Walked neutrally, avoided pulling | Pulling force measured with strain gauge<br><br>Stress behaviours (whale eye, looking at handler, barking, yelping, whining, howling, growling, lip licking, lowered tail, crouch, yawning, shake off, quivering, sniffing, balking) | - Dogs pulled most on a martingale collar<br>- Dogs exhibited more lip licking when wearing the harness compared to the martingale collar |
| Pauli et al. [47] | N: 26<br>Population: Pet<br>Weight: Not reported, but note breeds are all medium/large.<br>Breed: 12 Alaskan Malamute, 8 Siberian Husky, 4 American Staffordshire Terrier, 1 Chinook<br>Age: Not reported<br>Sex: Not reported | - Collar: Nylon (brand and model not specified)<br>- Harness: Nylon (brand and model not specified)<br><br>Picture: no | A force gauge was used to measure the tension each dog generated against the leash.<br>Then each dog was restrained while standing and the previously measured tension was replicated by pulling on the leash for 10 s.<br><br>Order: fixed (collar, harness). | Intraocular pressure measured before (baseline), 10 seconds after and 1 min after pulling | - Intraocular pressure increased significantly from baseline when pressure was applied while wearing a collar but not a harness |

| | | | | | |
|---|---|---|---|---|---|
| | | | Leash details not reported<br><br>Handler: Not reported. | | |
| Shih et al. [59] | N: 52<br>Population: Shelter<br>Weight: 16 to 43 kg (Median = 24 kg, interquartile range (IQR) = 7.76 kg).<br>Breed: Not reported<br>Age: 13 months – 11 years<br>Sex: 23 males, 29 females<br><br>Each dog had an assigned walking level:<br>1 (loose leash, n=1)<br>2 (occasional pulling, n=26),<br>3 (frequent pulling, n=21) 3+ (severe behavioural issues but not necessarily more pulling than level 3, n=4) | - Collar: Plain neck collar (various brands, not specified).<br>- Chest-strap harness: Black Dog Wear Pty Ltd "Balance Harness"<br><br>Picture: yes | Experimenter attracted the dog with a treat and walked away, placing it 50 cm in front of the dog when the leash reached full length<br><br>Within-subject counterbalanced design. Dogs wore both devices but only one was connected each time<br><br>Order: Randomized device, fixed first treats then toy. 10 min break.<br><br>1.4 leash was fixed to a tie-up ring secured to the wall. Attached at the front<br><br>Handler: N/A | Maximal and mean leash tension and proportion of pulling time. Measured with leash tension metre (RobacScience)<br><br>Stress behaviours (ears position, panting, lip licking, sniffing, shaking, paw lifting, tail position, vocalizing) and looking at the experimenter | - Dogs pulled harder and more steadily when the leash was connected to a harness compared to a collar and they were attracted with treats instead of a toy<br>- There was no significant increase in stress related behaviours<br>- Dogs looked more at the experimenter when wearing a harness in the food condition |
| Zilocchi & Parisi [67] | N: 18<br>Population: Pets<br>Weight: Not reported<br>Breed: Various breeds and mixes<br>Age: 1.5 to 12 years (5.5±1.2 year)<br>Sex: 12 females (9 spayed), 6 males (4 neutered) | - Collar: fixed nylon collar brand and model not reported<br><br>- Harness: type, brand or model not reported<br><br>Picture: no | Dogs met another dog in a fenced area (5m x 5m) while wearing both the collar and the harness. Free to interact while leashed for 1 min<br><br>Order: Randomized 1.5 m leash<br><br>Handler: Owner<br>Handler side: Not reported<br>Handler behaviour: Owner instructed not to communicate with the dog | Calming/ stress behaviours, social behaviours, aggressive behaviours and attention-seeking behaviour | - More lip licking when wearing the harness, and paw lifting when wearing the collar<br>- More attention-seeking signals towards the owner when wearing the collar<br>- Neither tool affected interspecific behaviour significantly |

**Supplementary Table S4.** Studies comparing head collars

| | Sample | Type of restraints used | Design | Measurements | Key findings |
|---|---|---|---|---|---|
| Haug et al. [61] | N: 12<br>Population: Pets<br>Weight: 16 - 36 kg | 4 types of head collar (models not specified):<br>- Gentle Leader | Sessions which consisted of 5 min of stationary handler (to | Behavioural measures grouped as | - No differences in either group of |

| | | | | | |
|---|---|---|---|---|---|
| | Breed: Not reported<br>Age: Not reported<br>Sex: 8 female, 4 male | - Halti<br>- Snoot Loop<br>- Response<br><br>Picture: yes | assess the dog's initial reaction to the restraint), 2 min walking, and 3 min stationary again (unleashed, to assess if they could remove the head collar)<br><br>Order: Balanced in 4 sequences (3 dogs per sequence). Sessions 2 weeks apart<br><br>Leash details not reported.<br><br>Handler identity: Same for all<br>Handler side: Left | Group 1: pawing without making contact with the collar or the leash, pawing nose, biting/pawing leash, opening mouth, rubbing face, shaking head.<br>Group 2: rearing up, rolling on the ground, balking, rushing forward | behaviours across head collars<br>- Marked reduction in overall reactions to the head collars over time, suggesting they adapted to it quickly |
| Ogburn et al. [60] | N: 26<br>Population: "Random source" dogs housed at the University (kenneled)<br>Weight: 12 - 24 kg<br>Breed: Not reported<br>Age: > 1 year<br>Sex: Not reported | Head collar (nylon, model not specified):<br>- Gentle Leader<br>- Promise<br>- Alpha-M.<br><br>Collar: nylon 1" traditional buckle (brand not specified)<br><br>Picture: Yes (drawing) | Each test lasted 20 min and included baseline physiological measurements, basic obedience and final physiological measurements<br>The exercise sequence was repeated 3 times and included: Walking 10 m, sitting 10 s, walking 10 m, sitting 10 s, turning clockwise, walking 10 m to measuring area, sitting 10 s<br><br>Balanced crossover: Matched pairs of dogs experienced both conditions in counterbalanced order, with each dog acting as its own control (1-2 days apart)<br><br>Leash 6 foot (1.82m).<br><br>Handler identity: Same for all<br>Handler side: Not reported | Physiological: Blood pressure, pulse rate, respiratory rate, pupil diameter, plasma ACTH and cortisol<br><br>Behavioural: During the obedience portion were: balking, biting or fighting the leash, rearing up, shaking head, biting or pawing the leash, pawing nose, attempts to remove the device, dragging behind handler, pulling ahead handler, moving with reluctance, taut leash, choking or coughing sounds, looking at handler, leash corrections, repositioning by hand ("made to sit").<br><br>At the end of each section: | - No significant differences in physiological responses<br>- Dogs wearing the collar required more repositioning (considered more "disobedient" and "unruly")<br>- Dogs wearing the head collar showed increased crouching, lowered head and ears, fought the leash more and pawed at the device |

| | | | | | |
|---|---|---|---|---|---|
| | | | | head position (up or down), tail position (wagging, up or tucked) ear position (up or down) and posture (lie, sit, stand, or crouch) | |

**Supplementary Table S5.** Studies comparing specialized equipment in working dogs

| | Sample | Type of restraints used | Design | Measurements | Key findings |
|---|---|---|---|---|---|
| Peham et al. [12] & Galla et al. [68]<br><br>(Note [68] is an abstract) | N: 8<br>Population: Guide dogs<br>Weight: 22.5 ± 10.7 (5.5 - 39.4) kilogram<br>Breed: 4 Labrador Retriever, 1 Golden Retriever, 1 German shepherd, 2 Retriever crosses<br>Age: 0.8 - 7 years<br>Sex: 6 female (5 spayed), male, 2 male (neutered) | 3 chest-strap harnesses made of leather (custom made):<br>- H1: Frame mobility restricted with loops.<br>- H2: Padded in the spine<br>- H3: Rigid connection with the frame<br><br>Picture: yes | Course including walking in a straight line, turning left, turning right, going upstairs and going downstairs. Each dog completed each course 5 times for each of the harnesses<br><br>Order: randomized<br><br>Handler: Same for all<br>Hadler side: Left | Pressure determined using sensor strips on both sides at the front, sternum, chest, shoulders and back (Peham et al., 2013)<br><br>Kinematic movement of the spine (Galla et al., 2013) | - In all harnesses the highest pressure was found on "sternum right". It was highest for H1, while pressures were smaller for H2 and H3.<br>- Highest pressure load on the sternum, while back regions were not heavily loaded<br>- Highest pressure on the right could be due to handler walking on the right side of the dog<br>- Harness 1 restricted lateral movement of the spine |
| Knights & Wiliams [39] | N: 13<br>Population: Assistance dogs in training<br>Weight: Median 29.25kg (IQR = 25.1-29.63)<br>Breed: Labrador, Golden or cross between the two<br>Age: 15 - 22 months<br>Sex: 7 females, 6 males | 3 harnesses:<br>- Y-shaped with B type handle (triangular shaped, fits laterally around the sternal chest strap)<br>- Y-shaped with A type handle (rectangular shaped, fits more upright onto the dorsal part of the harness)<br>- Chest-strap usual working harness of the dog, with A type handle | Each dog was tested using all four restraint devices across three trials walking in a straight line<br><br>Order: randomized Latin Square design<br><br>Handler: Usual handler of each dog<br>Handler side: Left | Thoracic limb stride length and room of movement of shoulder, elbow and carpal joint, measured using Kinovea TM 2-D kinematic analysis software | - Differences in locomotion when wearing a harness compared to just the collar<br>- Significant changes in thoracic limb stride length and restriction of room of movement with the harness featuring the B type handle |

| | | | | | |
|---|---|---|---|---|---|
| | | - Collar: flat leather (brand and model not specified)<br><br>Picture: yes | | | |
| Sandberg et al. [40]<br><br>(Note this is an abstract) | N: 5<br>Population: Not reported<br>Weight: 25 - 35 kg<br>Breed: Not reported<br>Age: 4 - 6 years<br>Sex: Not reported | - Custom-fit tactical harness (no further information provided)<br><br>- No harness | Dogs walking and trotting (no further information provided) | Sagittal plane (flexion–extension), transverse plane (internal–external rotation), and frontal plane (abduction–adduction) kinematics for the shoulder, elbow and carpus measured using 19 reflective skin markers using a 3-D motion system | - Wearing a tactical harness affected the gait of all joints of the forelimb. The elbow was affected in all three planes at both a walk and trot, while the shoulder was only affected at a walk |
| Weissenbacher et al. [34] | N: 12<br>Population: Certified guide dogs<br>Weight: 31.6 ± 4.2 kg (23 – 39 kg)<br>Breed: 9 Labrador Retrievers, 1 flat-coated Retriever, 1 curly-coated Retriever and 1 large poodle<br>Age: 5 ± 2 years (2-8)<br>Sex: 9 male, 3 female (all neutered/spayed) | - Chest-strap: with straight handle (usual harness the dog wore).<br>- Y-shaped: Krämer Pferdesport (Sugar dog, Joshua Reflective functional dog harness)<br><br>- Collar (brand and model not specified) | Dogs were lead back and forth on an area which included a pressure plate integrated onto the floor. Measurements were valid when the dog looked forward, did not overstride and walked at a steady pace. At least five valid trials were used<br><br>Dogs were tested with the collar and leash, and with each harness with a leash, with a straight handle and a curved handle. A light pull was used with the handle to simulate the usual working conditions of guide dogs<br><br>Order: Randomized<br><br>Leash information not specified<br><br>Handler: Same for all (sighted)<br>Handler side: Left | Maximum vertical force, vertical impulse and stride length. Ground reaction forces using a pressure plate on the ground (Zebris Medical FDM). | - No differences when comparing the collar and the harnesses on a leash<br>- Both harnesses increased hind right impulse, compensated by decreased front left impulse, regardless of the type of handle used<br>- Stride length was decreased with the Y-shaped harness when used with handles |

**Supplementary Table S6.** Studies focusing on leash tension

| | Sample | Type of restraints used | Design | Measurements | Key findings |
|---|---|---|---|---|---|
| Shih et al. [54] | N: 111<br>Population: Shelter<br>Weight: 24.43 (±6.65) kg.<br>Breed: Not reported<br>Age: 44.82 (±29.37) months<br>Sex: not reported<br><br>Each dog had an assigned walking level:<br>1 (loose leash)<br>2 (occasional pulling),<br>3 (frequent pulling)<br>3+ (severe behavioural issues but not necessarily more pulling than level 3) | Collar and harness (type, brand and model not specified).<br><br>Based on the picture provided they appear to be:<br>- Collar: fabric<br>- Chest-strap harness<br><br>Picture: yes | Walking to and from kennel (length/duration not specified)<br><br>1.4 m leash connected to the tension meter then attached to the collar and harness at the front.<br><br>Handler: 74 volunteers<br>Side and behaviour not reported | Maximal tension, mean tension, pulling frequency. Measured both for the dog and the handler using a leash tension meter (custom made measurement device including a load cell and a tri-axial accelerometer) | - Larger/heavier dogs exerted higher tension, but pulled less frequently than smaller/lighter dogs. This was mirrored by handler behaviour<br>- Young dogs pulled more frequently. This was also mirrored by handler behaviour<br>- Well behaved dogs pulled less. However, handlers did not respond with lower forces in this case |
| van Herwijnen et al. [69] | N: 24<br>Population: Pet<br>Weight: 22.5 ± 10.7 (5.5 - 39.4) kg<br>Breed: Various<br>Age: 0.5-10 years<br>Sex: 18 male, 6 female | Collar (flat, brand and model not specified)<br><br>Harness: reported as "standard harness" (brand and model not specified).<br><br>Picture: no | Food-distraction course: 12 m straight path with pieces of chicken placed at fixed positions on either side.<br>Complex zigzag object-distraction: 12 m zigzag path with balls, fake dogs, food bowls and odd-shaped objects.<br><br>Leash 1.5-2 m<br><br>Handler: Owner<br>Handler side: Not reported<br>Handler behaviour: instructed to guide the dog through the path without the dog touching the objects | Leash tension measured using IPOS Technology rein Sensor | - Owner-dog dyads differed in consistent ways<br>- Increased leash tension in the complex zigzag course suggesting it was harder to navigate |

**Supplementary Table S7.** Evidence-based findings on effects of different types of restraint equipment for walking dogs.

| Equipment / Effect of device was observed | Collar | Martingale collar | Head collar | Chest-strap harness | Y-shape harness | Front-clip harness | Other harnesses |
|---|---|---|---|---|---|---|---|
| Restricted elbow movement | Not tested | Not tested | Not tested | No [64][1]<br><br>Yes, in Cocker and Springer Spaniels compared to Y-shaped harness [36] | Yes, when used with front-clip (see next column) [64][1] | Yes, flexion and extension [64][1] [1] | Yes, extension with no-pull harness, and flexion with chest-plate and step-in harness [64][1]<br><br>Yes, at a walk and a trot wearing a "tactical" harness [38] |
| Restricted shoulder movement | Not tested | Not tested | Not tested | Yes, extension [64][1]<br><br>Yes, extension compared to no harness at a walk and a trot [30]<br><br>Yes, compared to Y-shaped harness and collars in Labradors and compared to Y-shaped harness in Staffordshire Bull Terriers and mixed breed dogs [36] | Yes, when used with front-clip (see next column) [64][1]<br><br>Yes, extension compared to no harness and chest-strap harness at a walk and a trot [30]<br><br>Yes, compared to no harness for 2 out of 3 dogs, especially when leashed [63] | Yes, flexion and extension [64][1] | Yes, flexion and extension with chest-plate harness and step-in harness, flexion with H-shaped harness, and extension with no-pull harness [64][1]<br><br>Yes, at a walk wearing a "tactical" harness [38] |
| Gait | Not tested | Not tested | Not tested | No gait differences between harness and no harness on treadmill/ground with no leash. One harness reduced pelvic limb step width on the treadmill with a leash; no | Shorter stride length compared to collar in Labradors and Springer Spaniels [36] | Not tested | Not tested |

| | | | | | | | |
|---|---|---|---|---|---|---|---|
| | | | | effect on step/stride length.<br>All pelvic limb measures reduced with harness vs. no harness on the ground with leash [35]<br><br>Shorter stride length compared to collar and Y-shaped harness in French bulldogs, compared to collar in Cocker Spaniels and compared to Y-shaped harnesses in mixed breed dogs [36]<br><br>No differences in maximum vertical force, vertical impulse and stride length compared to collar [32] | No differences in maximum vertical force, vertical impulse and stride length compared to collar [32] | | |
| Increased pressure on the body | Increased pressure on the neck [43, 62]<br><br>Increased ocular pressure [45]<br><br>Increased ocular pressure in brachycephalic (but not dolichocephalic) dogs while stationary, and in both types of dogs when exercising [44] | Not tested | Not tested | Not tested | Ocular pressure was not increased [44] | Not tested | Ocular pressure was not increased [44] |
| Physiological measures of stress | No differences between collar and head collar [58] [2]<br><br>Increased respiratory rate | Not tested | No differences between collar and head collar [58][2] | Not tested | Not tested | Not tested | Increased respiratory rate in brachycephalic dogs while exercising [44] |

| | | | | | | | |
|---|---|---|---|---|---|---|---|
| | in brachycephalic dogs while stationary or exercising [44] | | | | | | |
| Behavioural signs of stress | More paw lifting compared to harness [65] | Less lip licking compared to front-clip harness [56] | Increased crouching, lowered head and ears compared to collar [58] | No differences compared to collar [57] | No differences compared to collar [55] | More lip licking compared to martingale collar [56] | More lip licking compared to collar [65] |
| Other behaviours | More attention seeking behaviours towards humans, but no differences in intraspecific behaviour compared to harness [65] | No differences in looking at the handler and sniffing compared to front-clip harness [56][3] | More repositioning needed, more fighting the leash, and pawing at the device compared to collar [58] | Looked more at the experimenter in condition with food present compared to collar [57] | No differences in looking at the owner, sniffing, tracking, and stopping compared to collar [55] | No differences in looking at the handler and sniffing compared to martingale collar [56] | No differences in intraspecific behaviour compared to collar [65] |
| Pulling | Less than with chest-strap harness [57] | More than with front-clip harness [56] | Less than with collar [58] | More than with collar [57] | More than with collar [41] | Less than with martingale collar [56] | |

[1] Harnesses were compared to a flat collar in Dowdeswell & Churchill [66]. Note that in this study a H-shaped harness was called "Y-front" and a Y-shaped harness clipped at the front was called "front-clip".
[2] Physiological measures in Ogburn et al. [60] included blood pressure, heart rate, respiratory rate, pupil diameter, plasma ACTH, and cortisol levels.
[3] Of the 15 behaviours analyzed in Johnson & Wynne [58] only looking at handler, lip licking, and sniffing occurred with sufficient frequency for statistical comparisons.
